# *TP53* mutants and non-HPV16/18 genotypes are poor prognostic factors for concurrent chemoradiotherapy in locally advanced cervical cancer

**DOI:** 10.1038/s41598-021-98527-2

**Published:** 2021-09-28

**Authors:** Ikumi Kuno, Daisuke Takayanagi, Yuka Asami, Naoya Murakami, Maiko Matsuda, Yoko Shimada, Sou Hirose, Mayumi Kobayashi Kato, Masaaki Komatsu, Ryuji Hamamoto, Kae Okuma, Takashi Kohno, Jun Itami, Hiroshi Yoshida, Kouya Shiraishi, Tomoyasu Kato

**Affiliations:** 1 Department of Gynecology, National Cancer Center Hospital, 5-1-1 Tsukiji, Chuo-ku, Tokyo, 104-0045 Japan; 2 Division of Genome Biology, National Cancer Center Research Institute, 5-1-1 Tsukiji, Chuo-ku, Tokyo, 104-0045 Japan; 3 Department of Radiation Oncology, National Cancer Center Hospital, Tokyo, 104-0045 Japan; 4 Department of Diagnostic Pathology, National Cancer Center Hospital, Tokyo, 104-0045 Japan; 5 Division of Medical AI Research and Development, National Cancer Center Research Institute, Tokyo, Japan; 6 RIKEN Center for Advanced Intelligence Project, Cancer Translational Research Team, Tokyo, Japan

**Keywords:** Cervical cancer, Cancer genomics

## Abstract

Targeted sequencing for somatic mutations across the hotspots of 50 cancer-related genes was performed using biopsy specimens to investigate whether clinicopathological factors and genomic alterations correlated with prognosis in locally advanced cervical cancer. Seventy patients diagnosed with International Federation of Obstetrics and Gynecology (FIGO) stage III to IVA cervical cancer underwent radiotherapy or concurrent chemoradiotherapy at the National Cancer Center Hospital between January 2008 and December 2017. Mutations were detected in 47 of 70 [67% of cases; frequency of genetic alterations was as follows: *PIK3CA* (51%), *FBXW7* (10%), *PTEN* (7.1%), and *TP53* (5.7%)]. The Cancer Genome Atlas (TCGA) datasets showed a similar distribution of somatic mutations, but *PIK3CA* mutation frequency was significantly higher in our cohort than in TCGA datasets (*P* = 0.028). Patients with *TP53* mutation were significantly related to poor progression-free survival (PFS) (hazard ratio [HR] = 3.53, *P* = 0.042). Patients with tumor diameters > 70 mm were associated with poor prognosis (HR = 2.96, *P* = 0.0048). Patients with non-HPV16/18 genotypes had worse prognosis than those with HPV16/18 genotypes (HR = 2.15, *P* = 0.030). Hence, patients with locally advanced cervical cancer, *TP53* mutation, large tumor diameter, and non-HPV16/18 genotype were independently correlated with poor PFS, despite concurrent chemoradiotherapy.

## Introduction

Cervical cancer is the fourth most commonly diagnosed cancer, and the fourth leading cause of cancer-related deaths in women worldwide^1^. Cervical cancer is often caused by sexually transmitted infections with most human papillomavirus (HPV) types, especially high-risk HPV 16 and 18^2^. Although screening for cervical cancer has improved over the past decade, more than 20% of cervical cancer patients were identified as International Federation of Obstetrics and Gynecology (FIGO) stage III–IV at initial diagnosis^3^. Most patients with locally advanced cervical cancer are treated with concurrent chemoradiotherapy (CCRT)^4^. Recently, the response rate for CCRT has increased due to the progress of irradiation technology, and complete response has been achieved in approximately 75% of patients^5^. However, some patients have a poor response to CCRT^6^; therefore, they relapse early after treatment and have a poor prognosis^7^. Clinical trials for providing additional treatment after CCRT to verify the increment of treatment effect, aiming to improve the prognosis in these patients, are currently underway^8^. Additionally, therapeutic drugs have been approved by the US Food and Drug Administration (FDA) for advanced cancer that use an immune checkpoint inhibitor, such as pembrolizumab^9,10^, and an angiogenesis inhibitor, such as bevacizumab^11,12^. However, the potential of benefiting from such molecular-targeting drugs is not well evaluated for patients with locally advanced cervical cancer.

In addition to tumor volume, performance status, treatment received, and prognostic factors for locally advanced cervical cancers, such as age, race, stage, histological type, grade, lymph node enlargement, and location, are associated with poor outcomes^4^. Several studies have focused on the prognostic factors for patients receiving CCRT in locally advanced cervical cancer, and it has been reported that larger tumor size and high-risk HPV were correlated with prognosis^13,14^. However, most studies have targeted operable cases. It is necessary to conduct a study on inoperable cases of locally advanced cervical cancer of FIGO stages III to IVA.

Comprehensive profiles of genomic alterations in cervical cancer have been published by The Cancer Genome Atlas (TCGA)^15^. *PIK3CA* mutations are one of the most frequently detected mutations in cervical cancer regardless of ethnicity^15–17^. Several studies have reported that cervical cancer patients with *PIK3CA* mutations are associated with worse prognosis than those without the mutation^17–19^. However, the results of some reports differ from these findings^16,20^, necessitating further discussion. In addition, most studies have focused on the association of single or multiple genetic mutations with prognosis using surgical specimens^9,17,19–21^; there are only a few reports on targeted sequencing using biopsy specimens from inoperable advanced cervical cancer^22^. Further studies are needed to elucidate the distribution of genomic alterations in locally advanced cervical cancer in order to identify novel therapeutic targets and biomarkers associated with prognosis. If poor prognostic factors or actionable mutation frequency are well understood, patients may opt for additional treatment after CCRT. Many previous studies have investigated the association of somatic mutations or clinicopathological factors, including high-risk HPV genotypes, with prognosis or response to adjuvant CCRT in early-stage cervical cancer patients receiving surgical operation. Only a few studies have suggested that these clinicopathological factors are associated with response to chemoradiotherapy in patients with locally advanced cervical cancer receiving CCRT.

In this study, we evaluated the pattern of genomic or actionable mutations in patients with FIGO stage III to IVA cervical cancer who received CCRT. We examined whether clinicopathological factors, including HPV genotypes and genomic alterations, were associated with prognosis in patients receiving CCRT.

## Results

### Patient characteristics

The selection flowchart for the 70 patients used in this study is shown in Figure S1, and the patient characteristics are summarized in Table 1. Of the 70 patients, 65 (93%) were diagnosed with squamous cell carcinoma (SCC), and 68 cervical tissue samples (97%) were HPV-positive. Thirty-three patients (47%) had lymph node enlargement, and were suspected to have pelvic and/or para-aortic lymph node metastases. Fifty-five (79%) patients underwent CCRT, and the standard concurrent chemotherapy regimen was cisplatin-based. Patients over seventy-five years of age or those who had difficulty receiving chemotherapy according to the physician’s choice only underwent RT. The median tumor size was 52.5 mm (range, 30–100 mm). The tumor diameter was over 70 mm in 16 (23%) patients. The median follow-up period was 54 months (range, 6–135 months). Two-year overall survival (OS), progression-free survival (PFS), and locoregional relapse-free survival (LRFS) were 77.1% (95% confidence interval [CI], 65.4–85.3), 60.0% (95% CI, 47.6–70.4), and 71.2% (95% CI, 58.9–80.3), respectively.

**Table 1:** Characteristics of locally advanced cervical cancer patients.

Variable	n	(%)
Total patients	70	
Age, median years [range]	63.5 [32–89]	
**FIGO stage (2018)**		
IIIA	6	(8.6)
IIIB	26	(37.1)
IIIC1r	18	(25.7)
IIIC2r	9	(12.9)
IVA	11	(15.7)
**Histology**		
Squamous cell carcinoma	65	(92.9)
Adenocarcinoma	3	(4.3)
Adenosquamous carcinoma	1	(1.4)
Neuroendocrine carcinoma	1	(1.4)
**HPV genotype**		
Positive	68	(97.1)
HPV16	22	(31.4)
HPV18	17	(24.3)
HPV31	10	(14.3)
HPV33	2	(2.9)
HPV45	1	(1.4)
HPV52	8	(11.4)
HPV58	3	(4.3)
HPV59	1	(1.4)
HPV82	1	(1.4)
HPV genotype not identified	3	(4.3)
Negative	2	(2.9)
**Lymph node enlargement**		
No	37	(52.9)
Yes	33	(47.1)
**Treatment**		
Radiation therapy	15	(21.4)
Concurrent chemoradiotherapy	55	(78.6)
**Tumor size (mm) (median [range])**	52.5 [30–100]	
< 70	54	(77.1)
≥ 70	16	(22.9)
Median follow-up period, month [range]	53.5 [6–135]	

### Correlation between clinicopathological factors and prognosis

We examined whether previously reported clinicopathological factors were correlated with prognosis in locally advanced cervical cancer. Univariate and multivariate analyses revealed that a tumor diameter larger than 70 mm was an unfavorable prognostic factor, especially for PFS (HR = 2.96, *P* = 0.0048) (Table 2, Table S1).

**Table 2:** Correlation between clinico-pathological factors and progression free survival in locally advanced cervical cancer patients.

Variable	Univariate		Multivariate*	
	Hazard ratio	*P* value	Hazard ratio	*P* value
Age (≥ 60/< 60)	1.08 (0.54–2.15)	0.84	0.92 (0.43–1.98)	0.84
FIGO Stage (IV/III)	0.74 (0.29–1.90)	0.53	0.59 (0.22–1.60)	0.30
Histology (non-SCC/SCC)	3.38 (1.29–8.88)	0.013	2.70 (0.97–7.50)	0.057
Lymph node enlargement (positive/negative)	1.56 (0.82–2.99)	0.18	1.36 (0.68–2.74)	0.38
Treatment (RT**/CCRT***)	1.48 (0.73–2.99)	0.28	1.83 (0.84–3.97)	0.13
Tumor size (≥ 70 mm/< 70 mm)	2.49 (1.24–5.00)	0.01	2.96 (1.39–6.29)	0.0048

*Cox proportional hazards regression analysis, **Radiation therapy, ***Concurrent chemoradiotherapy.

### Comparison of somatic mutation patterns in locally advanced cervical cancer using the present and TCGA datasets

**Figure 1:**
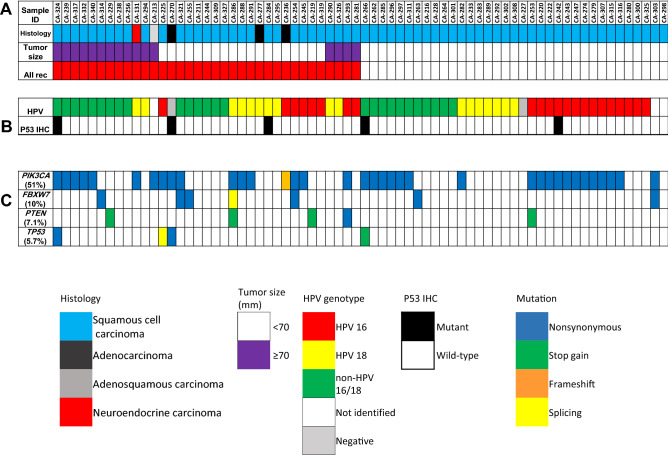
Clinicopathological factors and mutation profile (more than 5% frequency) in our cohort. (**A**) Clinical factors, histological types, and recurrence status; (**B**) HPV genotype and IHC staining pattern; and (**C**) mutation profile of the seventy patients with cervical cancer. Mutated genes are color-coded according to their mutation type. Data analysis was carried out using the Torrent Suite Software v5.0.4 (Thermo Fisher Scientific).

Targeted sequencing for the 70 specimens of locally advanced cervical cancer revealed pathogenic/oncogenic mutations in 47 cases (67%). *PIK3CA* was the most frequent genomic alteration detected in this study, with a frequency of 51%, followed by *FBXW7* (10%), *PTEN* (7.1%), *RB1* (13%) and *TP53* (5.7%) (Fig. 1). There was no association between genomic mutation frequency and tumor size or lymph node enlargement. Twelve patients had local recurrence, ten of whom (83%) had pathogenic/oncogenic mutations. Further, the frequency of genomic alterations in patients with stage III to IVA cervical cancer in TCGA dataset was as follows: *PIK3CA* (30%), *FBXW7* (15%), *PTEN* (15%), and *TP53* (11%) (Figure S2)*.* The mutation rates were 65% (42 of 65) in SCC and 100% (5 of 5) in non-SCC cases. The somatic mutations of locally advanced cervical cancer in TCGA datasets are summarized in Figure S2. There was no statistical difference in the distribution of somatic mutations between our cohort and TCGA datasets, except for the frequency of *PIK3CA* mutation in our cohort, which was higher than that in TCGA datasets (*P* = 0.028, Table S2).

### Actionable mutation in locally advanced cervical cancer

Actionable mutations registered with evidence levels of 1–3B in OncoKB were detected in 35 of 70 (50%) patients (Figure S3). Most somatic mutations were dominated by *PIK3CA* mutation, which is similar to TCGA datasets (Figure S3). These results indicated that 30–51% of patients with locally advanced cervical cancer may have benefited from mTOR/AKT/PI3K inhibitors.

### Correlation between genomic alteration and prognosis

**Figure 2:**
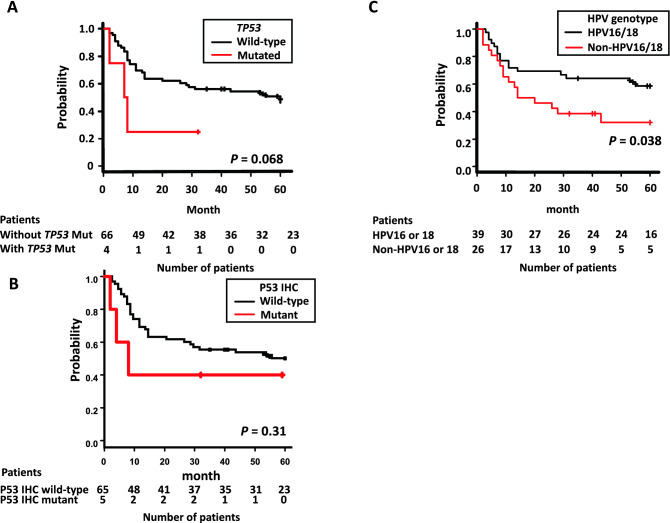
Kaplan–Meier survival curves according to *TP53* status and HPV genotypes. (**A**) Progression-free survival between *TP53* wild-type (black line) and *TP53* mutants (red line), (**B**) Progression-free survival between *TP53* wild-type of IHC (black line) and *TP53* mutant of IHC (red line), and (**C**) Progression-free survival between HPV16/18-positive (black line) and non-HPV16/18 (red line) patients.

Genomic alterations in *PIK3CA*, *FBXW7*, and *PTEN* were not significantly correlated with PFS (Table S3). Although all patients with *TP53* mutation (n = 4) received CCRT, and the tumor diameter was less than 70 mm in three out of four cases, *TP53* mutants were independently correlated with poor survival (Fig. 2A). Most patients experienced recurrence within one year after the start of RT. Multivariate Cox proportional regression analysis indicated that patients with *TP53* mutations were associated with poor PFS (HR = 3.53, *P* = 0.042, Table 3A, Table S4).

**Table 3:** Correlation between *TP53* mutation or HPV genotypes and progression-free survival in locally advanced cervical cancer patients.

Variable	Univariate		Multivariate*	
	Hazard ratio	*P* value	Hazard ratio	*P* value
**(A) *TP53* mutation (n = 70)**				
Lymph node enlargement (Positive/negative)	1.56 (0.82–2.99)	0.18	–	
Treatment (RT**/CCRT***)	1.48 (0.73–2.99)	0.28	–	
Tumor size (≥ 70 mm/< 70 mm)	2.49 (1.24–5.00)	0.01	2.69 (1.33–5.44)	0.0060
*TP53* mutation (n = 4)	2.85 (0.86–9.44)	0.061	3.53 (1.05–11.92)	0.042
**(B) Non-HPV16/18 (n = 65)**				
Lymph node enlargement (positive/negative)	1.56 (0.82–2.99)	0.18	–	
Treatment (RT**/CCRT***)	1.48 (0.73–2.99)	0.28	–	
Tumor size (≥ 70 mm/< 70 mm)	2.40 (1.17–4.96)	0.018	2.37 (1.14–4.93)	0.021
HPV genotype (non HPV16 or 18/HPV16 or 18)	2.17 (1.10–4.29)	0.026	2.15 (1.08–4.27)	0.030

*Stepwise multiple regression analysis, **Radiation therapy, ***Concurrent chemoradiotherapy.

### Association of p53 status with poor outcomes

We investigated the protein expression of p53 by IHC staining of the specimens of 70 patients. Representative results of IHC staining are shown in Figure S4. Five specimens presented with mutant p53 staining patterns, including three with *TP53* mutations. p53 mutant staining patterns tended to be associated with poor survival of patients when compared with wild-type staining patterns (Fig. 2B).

### Non-HPV16/18 patients had poorer survival than HPV16/18 patients

Sanger sequencing identified 65 cases of HPV genotypes, and we divided the HPV-positive patients into two groups—HPV16 or 18 (HPV16/18) group and non-HPV16/18 group. We investigated the correlation of the HPV16/18 and non-HPV16/18 groups with prognosis. Univariate and multivariate analyses showed that the non-HPV16/18 group had lower PFS compared with the HPV16/18 group (HR = 2.15, *P* = 0.030; Fig. 2C, Table 3B, Table S5). The non-HPV16/18 group was older than the HPV16/18 group, and the percentages of people over the age of 60 in both were 81% and 51% (*P* = 0.020), respectively. Owing to older age, more patients without HPV16/18 only received RT (15% vs. 31%, *P* = 0.22). Tumor diameter in the non-HPV16/18 group was higher than that in the HPV16/18 group; however, the difference was not significant. The percentage of patients whose tumor diameter was greater than 70 mm was 15% in the HPV16/18 group and 35% in the non-HPV16/18 group (*P* = 0.13).

## Discussion

In this study, we identified the mutation profile and prognostic factors in patients with inoperable locally advanced cervical cancer who received CCRT or RT. Thirty to fifty percent of patients with locally advanced cervical cancer might have benefited from molecular-targeting drugs. Further, *TP53* mutation, large tumor size, and non-HPV16/18 genotypes were indicative of poor prognosis.

In line with previous reports^5,23^, patients with tumor diameters over 70 mm had poor PFS in our cohort. In a phase II study of CCRT with brachytherapy in Japanese patients with locally advanced cervical cancer (JGOG1066)^5^, tumor diameter was correlated with PFS. In a prospective study, it was reported that a tumor diameter of ≥ 60 mm was an independent poor prognostic factor^23^. In our cohort, tumors ≥ 60 mm and ≥ 70 mm had an HR of 2.46 (95% CI, 1.28–4.71) and 2.49 (95% CI, 1.24–5.00) in univariate analysis, respectively. This suggests that the therapeutic effects of CCRT in patients with locally advanced cervical cancer are limited when the tumor diameter exceeds 60–70 mm. Therefore, these patients need additional treatments, such as molecular-targeting drugs based on actionable mutations.

In the past decade, next-generation sequencing has been performed for operable cervical cancer in patients receiving adjuvant CCRT or RT, and many genomic alterations have been identified. Although some genomic alterations, such as *PIK3CA*^17,19,21^, *KRAS*^24^, *ERBB2*^25^, and *STK11*^16^, have been reported to correlate with prognosis, these mutations were not statistically associated with prognosis in our cohort of patients with locally advanced cervical cancer. In our cohort, among the somatic mutations identified in more than 5% cases, patients with *TP53* mutation showed poor prognosis. Three out of four patients with *TP53* mutation showed recurrence after CCRT within 10 months, and the tumors with this mutation were aggressive. Many studies suggest that *TP53* regulates malignant phenotypes by gain-of-function mutations, including mutations detected in this study. A previous report showed that *TP53* mutants had the worst OS when compared with wild-type or deletion-type *TP53* variants^26^. Further, E6 and E7 oncoproteins secreted by high-risk HPV were expressed consistently. E6 and E7 proteins form complexes with p53 and retinoblastoma (Rb), respectively, and inhibit the activation of proteins in cell cycle regulation^27^. Interpretation of p53 IHC staining in cervical carcinoma has not been formally established^26,28^. In this study, we evaluated p53 IHC staining patterns as wild-type or mutant patterns using the p53 IHC staining patterns previously reported for vulvar SCC^29^. Three out of four patients with *TP53* mutations had mutant type IHC staining pattern; patients with p53 IHC mutant type tended to be associated with poorer survival than the wild-type. These results indicate that p53 IHC staining can be used to evaluate cervical cancer. Although the correlation between *TP53* mutation and radiosensitivity in patients with cervical cancer has not been fully studied, some studies have shown that *TP53* mutation is correlated with poor prognosis after RT in SCC of the head and neck^30–32^. *TP53* is the most frequently mutated gene across all cancer types^33^. *TP53* mutation may also be associated with radioresistance or poor prognosis in cervical cancer.

In our study, patients without HPV16/18 had poorer PFS than those with HPV16/18. Many previous studies have shown that early-stage cervical cancer patients with HPV16 and HPV18 have worse prognosis than patients without HPV16/18^34^. On the other hand, there are several conflicting results regarding the association between HPV type and survival due to differences in radiotherapy. Recent studies have reported better survival of patients with HPV16-positive or HPV16/18-positive genotypes who were administered CCRT for FIGO stage III/IV tumors^35–37^. Therefore, non-HPV16/18 status might be a poor prognostic factor, as it changes the response to chemotherapy. Although few reports have focused on the correlation between HPV genotypes and p53 status, non-HPV16/18 status might affect prognosis by exhibiting marked alterations in p53. It is also important to prevent cervical cancer caused by high-risk HPVs other than HPV16/18. A 9-valent HPV vaccine has been developed, which will provide protection against non-HPV16/18 infection and advanced tumors.

Using targeted sequencing, genomic alterations, such as *PIK3CA*, linked to molecular-targeting drugs were detected in locally advanced cervical cancer in both our cohort and TCGA dataset. *PIK3CA* is the most frequently mutated gene, playing a key role in the growth and differentiation of HPV-immortalized cells^38^. In addition, activation of the PI3K/AKT/mTOR pathway through *PIK3CA* regulates various transformed phenotypes^38^. The PI3K inhibitor alpelisib has been approved for treatment of hormone receptor-positive and human epidermal growth factor receptor 2-negative breast cancer by the US FDA^39^. Notably, therapeutic benefit from this drug has been observed in three of five cervical cancer patients harboring *PIK3CA* mutation in a phase I trial^40^. Therefore, this drug is a promising therapeutic option for locally advanced cervical cancer. There are several limitations in our study. First, this study was a single institution retrospective study and the number of cases participating this study was limited. Second, targeted sequencing for mutation analysis was performed in this study; therefore, we could analyze only hotspot mutations in 50 cancer-related genes. We will further perform genomic analysis for predicting prognosis of cervical cancer patients and outcomes of targeted therapies.

In conclusion, we presented the profile of genomic alterations of locally advanced cervical cancer in both our cohort and TCGA dataset. We identified that *TP53* mutants were correlated with poor PFS in locally advanced cervical cancer. In addition, tumors with diameter greater than 70 mm and non-HPV16/18 genotype were associated with poor survival. Actionable mutations for molecular-targeting drugs were detected in more than half of our cohort. These prognostic factors may lead to the development of novel treatment approaches for patients with locally advanced cervical cancer.

## Materials and methods

### Patients and tumor samples

One hundred and thirteen patients underwent RT or CCRT at the National Cancer Center Hospital, Tokyo, between January 2008 and December 2017 (Figure S1). Seventy of the 113 Japanese patients had locally advanced cervical cancer with FIGO stage IIIA to IVA, and these patients were recruited for this study. Patients received external beam RT and brachytherapy^41^, and most of the chemotherapy regimen was cisplatin-based. Clinicopathological data, including age at histological diagnosis, FIGO stage, histological subtypes, status of pelvic/para-aortic lymph nodes, tumor size, treatment, and follow-up, were obtained from the electronic medical records. Cervical tumor specimens were collected by punch biopsy of the tumor before CCRT. The specimens were fixed in 10% neutral buffered formalin and embedded in paraffin (FFPE).

### Treatment regimens for CCRT and RT

All patients, except one, received both external beam RT and brachytherapy (intracavitary brachytherapy or intracavitary/interstitial brachytherapy). The initial 20–40 Gray (Gy) was delivered to the whole pelvis using the 4-field box technique, followed by a 40 mm-wide midline block until pelvic side wall dose of 50 Gy. If enlarged lymph nodes were present, an additional 6–10 Gy was delivered with smaller fields. After the initiation of the midline block, a total of 3–4 sessions of brachytherapy were performed in 1–2 sessions per week, and the dose per fraction was 6 Gy. All brachytherapy was performed by an ^192^Iridium remote afterloading system (RALS, MicroSelectron, HDR™, Elekta, Veennendaal, The Netherlands). The concurrent chemotherapy regimen was usually 40 mg/m^2^/week of cisplatin, whereas some patients received other regimens, such as carboplatin, cisplatin plus tegafur, gimeracil, oteracil, and cisplatin plus fluorouracil.

### DNA preparation and next-generation sequencing

Genomic DNA was extracted from FFPE tumor tissues using the QIAamp DNA FFPE tissue kit (Qiagen, Hilden, Germany), according to the manufacturer’s instructions. Purified genomic DNA (50 ng) obtained from tumor tissues was used for library construction using the Ion AmpliSeq™ Cancer Hotspot Panel v2 (Thermo Fisher Scientific, Waltham, MA, USA), which targets approximately 2800 COSMIC mutational hotspot regions of 50 cancer-related genes. An Ion AmpliSeq™ Custom Panel, designed for the *TP53* gene (coverage: all coding regions) using Ion AmpliSeq™ Designer (https://www.ampliseq.com), was also used. Sequencing was performed on the Ion Proton platform (Thermo Fisher Scientific). For quality control, samples with a mean read depth of coverage over 1000 and a base quality score of 20 (with ≤ 1% probability of being incorrect), which accounted for 90% of the total reads, were selected.

### Locally advanced cervical cancer in TCGA database

We selected 54 cases with locally advanced cervical cancer registered in TCGA database. Somatic mutations called from whole genome sequencing and whole exome sequencing data available in TCGA database were downloaded as a mutation annotation format (MAF) file via the cBioPortal for Cancer Genomics (http://www.cbioportal.org).

### Classification of oncogenic/pathogenic mutations

Data analysis was carried out using the Torrent Suite Software v5.0.4 (Thermo Fisher Scientific). We selected mutations that met the following criteria: the frequency of variant alleles was more than 4% in tumor tissues; single nucleotide polymorphisms were excluded if they showed a threshold allele frequency ≥ 0.01 in either the National Heart, Lung, and Blood Institute (NHLBI) Grand Opportunity Exome Sequencing Project (ESP6500; http://evs.gs.washington.edu/EVS/) or the integrative Japanese Genome Variation Database (iJGVD, 20181105; https://ijgvd.megabank.tohoku.ac.jp/). The variants have been registered as “pathogenic/likely pathogenic variants” in ClinVar^42^ or “oncogenic/likely oncogenic variants” in OncoKB (http://oncokb.org) databases using the OncoKB annotator commit 8910b65 (accessed on June 29, 2019). All selected variants were validated using the Integrative Genomics Viewer (IGV; http://www.broadinstitute.org/igv/).

### Definition of actionable mutations

OncoKB is a precision oncology knowledge database that contains information on the effects and treatment implications of specific genomic alterations in cancer patients. Somatic mutations and copy number alterations have been categorized into four evidence levels. In the present study, genetic aberrations with evidence levels 1–3B according to OncoKB level of evidence V2 were designated as actionable mutations for molecular-targeting drugs^43^.

### Immunohistochemical (IHC) staining of p53

IHC staining was performed on FFPE specimens. Representative whole 4 μm-thick sections were analyzed. After deparaffinization, the protein expression of p53 was evaluated using a monoclonal antibody against human p53 protein (clone DO-7, Dako, Glostrup, Denmark). IHC staining was performed using a Dako autostainer (Dako, CA, USA) and visualized using EnVision Detection System (Dako), according to the manufacturer’s instructions. The slides were counterstained with hematoxylin. Staining for p53 expression was evaluated as wild-type or mutant^29^. Scattered, mosaic, mid-epithelial p53 expression was considered to represent the wild-type staining pattern. Mutant staining pattern was characterized by diffuse strong nuclear positivity in the basal and upper layers of the tumor cells, or complete absence of p53 staining with appropriate positive internal control.

### Identification of HPV genotyping by Sanger sequencing

HPV genotyping was performed for the 70 cases. Genomic DNA (10 ng) was amplified via polymerase chain reaction (PCR) using TaKaRa Taq DNA polymerase (Takara Bio Inc., Shiga, Japan) for two distinct HPV genomic regions. The HPV E6/E7 homologous region was amplified using the pU-1M/pU2R (HPVpU-1M: 5′-TGTCAAAAACCGTTGTGTCC-3′, and HPVpU-2R: 5′-GAGCTGTCGCTTAATTGCTC-3′) primer set, and the region containing the HPV L1 gene was amplified using the GP5+/GP6+ (GP5+: 5′-TTTGTTACTGTGGTAGATACTAC-3′, and GP6+: 5′-GAAAAATAAACTGTAAATCATATTC-3′) primer set. PCR reactions were performed using the TaKaRa PCR Human Papillomavirus Typing Set (TakaRa Bio Inc.). PCR products were purified using the NucleoSpin Gel (Takara Bio Inc.) or PCR Clean-up kit (Takara Bio Inc.). Sanger sequencing was performed using an ABI 3130xl DNA Sequencer (Applied Biosystems, Foster City, California, USA), according to the manufacturer’s instructions. Similarity between the obtained sequences and various HPV genotypes in the GenBank database was determined using Basic Local Alignment Search Tool (BLAST) (https://blast.ncbi.nlm.nih.gov/Blast.cgi).

### Detection of high-risk HPV types in cervical cancer tissues

To clarify the frequency of HPV-positive results in these samples, we performed in situ hybridization assay for HPV detection (HPV-ISH) using HPV-III High Risk probes (Roche Diagnostics, Mannheim, Germany), according to the manufacturer’s instructions. This assay can detect high-risk HPV genotypes, including HPV-16, 18, 31, 33, 35, 45, 52, 56, 58, and 66, in cervical cancer specimens^16^.

### Statistical analysis

The Kaplan–Meier method was applied to estimate survival, PFS, and LRFS. Differences in outcomes were compared using the log-rank test. PFS was defined as the interval from the start of the first RT to either disease progression or death. OS was defined as the interval from the start of the first RT to death. LRFS was defined as the interval from the start of first RT to either locoregional disease progression or death. PFS, OS, and LRFS were determined at the last contact date for each patient. Cox regression analysis was used to assess the univariate prognostic significance of survival. Using multivariate Cox proportional-hazards models, we considered each mutation status, histological subtype, para-aortic lymph node metastasis, and tumor size. The data cut-off date was January 29, 2020. Statistical analyses were performed with EZR version 1.37^44^, which is based on R and R commander.

### Ethics declarations

This retrospective study was approved by the Institutional Review Board of National Cancer Center Hospital (approval number 2017-136) and follows the ethical standards laid down in the Declaration of Helsinki. Informed consent was obtained from all patients.

## Supplementary Information

Supplementary Figures.

Supplementary Figure 4.

Supplementary Legends.

Supplementary Tables.
