# Supplementary figures and images for: TP53 mutants and non-HPV16/18 genotypes are poor prognostic factors for concurrent chemoradiotherapy in locally advanced cervical cancer
Source: Sci Rep. 2021 Sep 28;11:19261. doi: 10.1038/s41598-021-98527-2 (PMC8478905; doi:10.1038/s41598-021-98527-2)

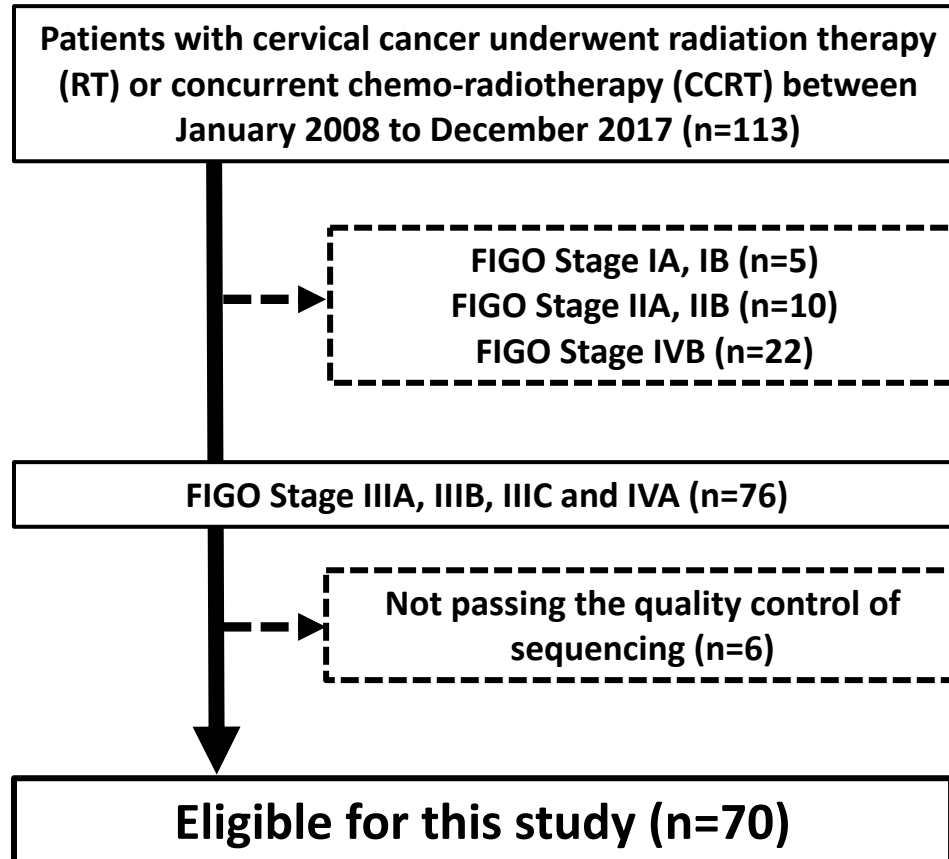

**Figure S1**



(A) Our cohort (n=70)

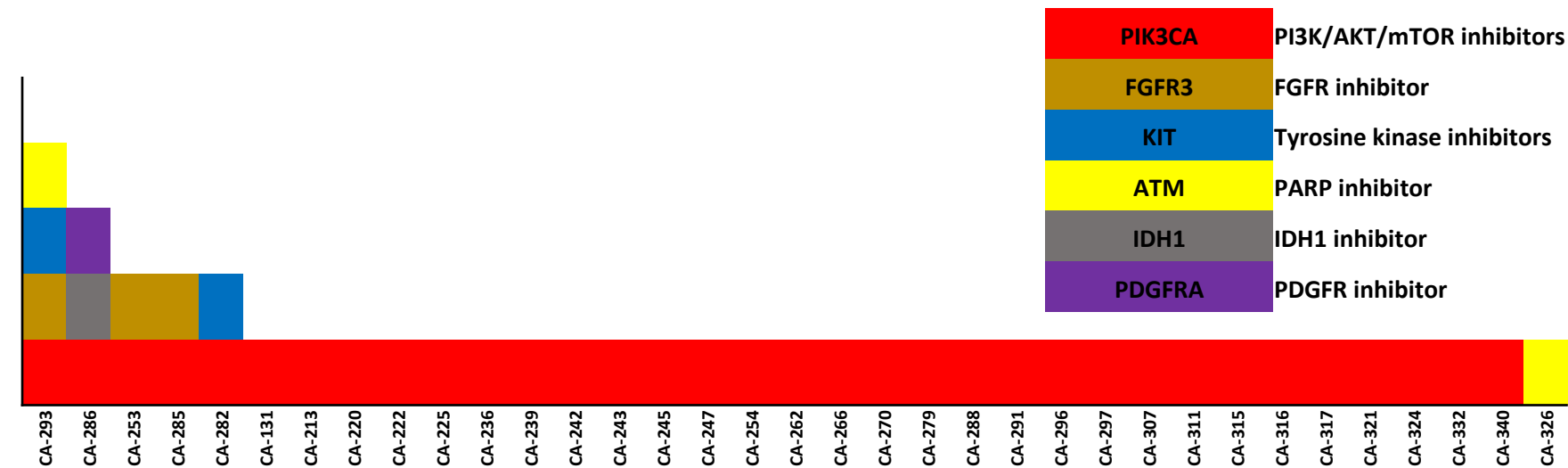

(B) TCGA datasets (n=54)

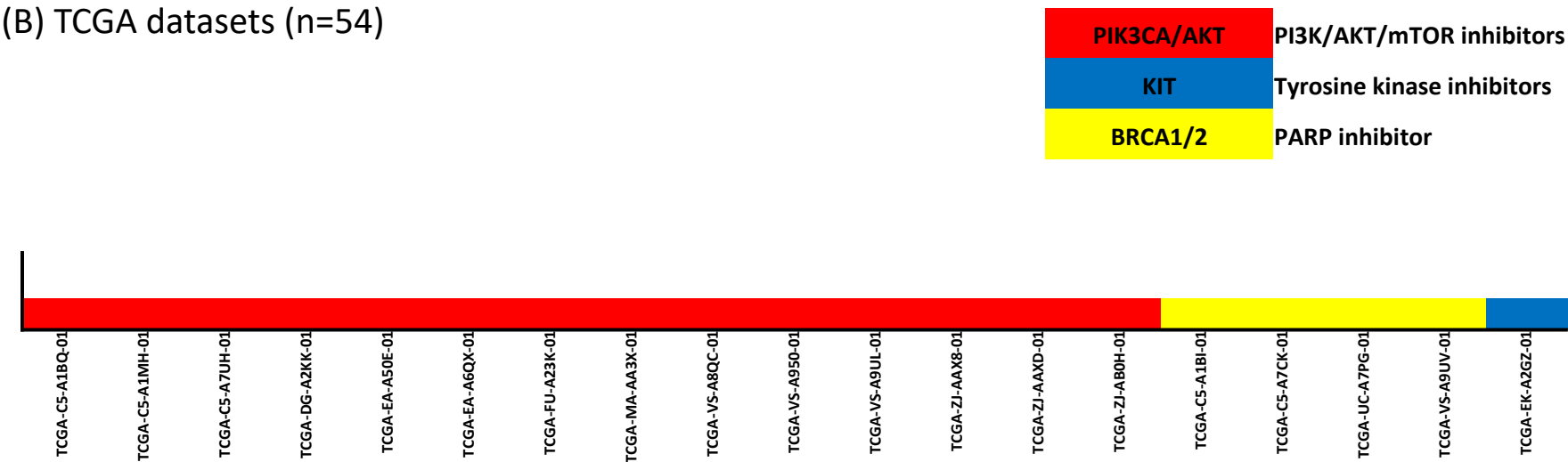

**Figure S3**

Supplement: Supplementary file 1 — Supplementary Figures. [file 41598_2021_98527_MOESM1_ESM.pdf]
