# Supplementary material for: TP53 mutants and non-HPV16/18 genotypes are poor prognostic factors for concurrent chemoradiotherapy in locally advanced cervical cancer
Source: Sci Rep. 2021 Sep 28;11:19261. doi: 10.1038/s41598-021-98527-2 (PMC8478905; doi:10.1038/s41598-021-98527-2)
Supplement: Supplementary file 2 — Supplementary Figure 4. [file 41598_2021_98527_MOESM2_ESM.pdf]

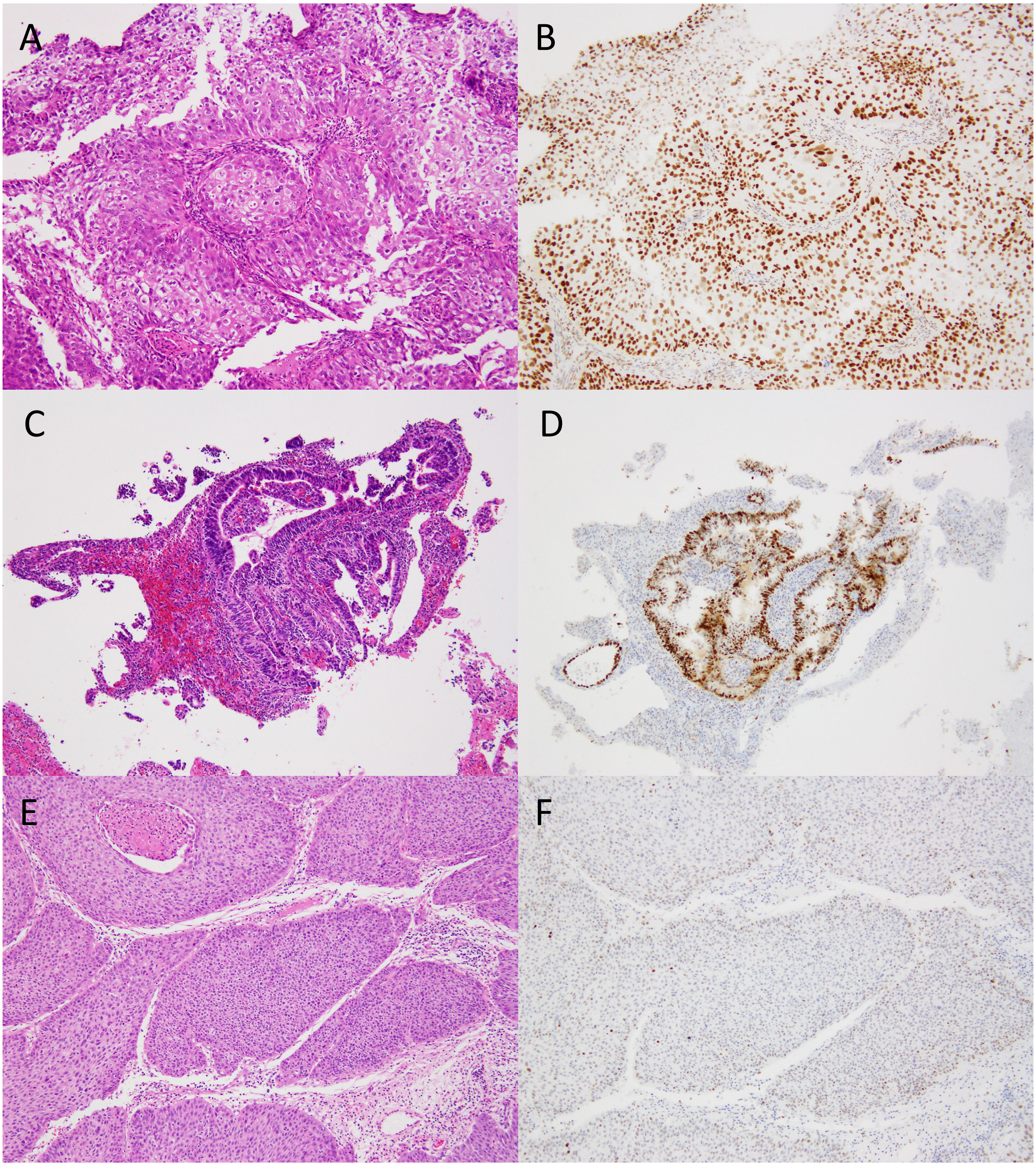

Supplementary Figure 4. Representative microphotographs of p53 immunohistochemistry in cervical cancer. Squamous cell carcinoma with *p53* gene mutation (A, CA#324) show diffuse strong positivity of p53 (B). Endocervical adenocarcinoma with *p53* gene mutation (C, CA#270) present diffuse strong positivity of p53 (D). Squamous cell carcinoma without *p53* gene mutation (E, CA#325) show scattered p53 positive cells, so-called wild-type staining pattern (F). A, C, and E (H&E, x100); B, D, and F (p53, x100).
