# Supplementary material for: TP53 mutants and non-HPV16/18 genotypes are poor prognostic factors for concurrent chemoradiotherapy in locally advanced cervical cancer
Source: Sci Rep. 2021 Sep 28;11:19261. doi: 10.1038/s41598-021-98527-2 (PMC8478905; doi:10.1038/s41598-021-98527-2)
Supplement: Supplementary file 3 — Supplementary Legends. [file 41598_2021_98527_MOESM3_ESM.docx]

**Supplementary material**

Figure S1. Flowchart for patient selection

Figure S2. Clinicopathological factors and mutation profile (more than 5% frequency) in TCGA dataset

Figure S3. Fraction of patients with actionable mutation for molecular-targeting drugs

Figure S4. Representative microphotographs of p53 immunohistochemistry in cervical cancer
